# The rs2233678 Polymorphism in PIN1 Promoter Region Reduced Cancer Risk: A Meta-Analysis

**DOI:** 10.1371/journal.pone.0068148

**Published:** 2013-07-09

**Authors:** Qi Li, Zhao Dong, Yun Lin, Xinyan Jia, Qun Li, Hong Jiang, Liwei Wang, Yong Gao

**Affiliations:** 1 Department of Oncology, Shanghai First People’s Hospital Affiliated Shanghai Jiaotong University, Shanghai, China; 2 Department of Radiotherapy, No.85 Hospital of People’s Liberation Army, Shanghai, China; 3 Department of Oncology, Shanghai East Hospital, Tongji University, Shanghai, China; Winship Cancer Institute of Emory University, United States of America

## Abstract

**Background:**

Published evidence suggests that the rs2233678 (−842 G>C) polymorphism in the PIN1 (peptidyl-prolyl cis/trans somerase NIMA-interacting 1) promoter region may be associated with cancer risk; however, the conclusion is still inconclusive.

**Methods:**

We conducted a meta-analysis to determine whether −842 G>C polymorphism was associated with cancer risk. Odds ratio (OR) and 95% confidence intervals (95% CI) were used to assess the strength of association. Genotype distribution data and adjusted ORs were collected to calculate the pooled ORs. Meta-regression was conducted to detect the source of heterogeneity. Publication bias was evaluated by Egger’s test and Begg’s test.

**Results:**

A total of 11 eligible studies, including 9280 participants, were identified and analyzed. Overall, we found that carriers of the −842 C allele were associated with significantly decreased cancer risk (C vs. G, OR = 0.750, 95% CI: 0.639–0.880, P_heterogeneity_ = 0.014, estimated by genotype distribution data; CC+GC vs. GG, OR = 0.668, 95% CI: 0.594–0.751, P_heterogeneity_ = 0.638, estimated by adjusted ORs). No evidence of publication bias was observed. Meta-regression revealed that ethnicities (p = 0.021) and sample size (p = 0.02) but not sources of control (p = 0.069) were the source of heterogeneity.

**Conclusion:**

These results suggest that the PIN1 rs2233678 (−842 G>C) polymorphism significantly reduces cancer risk.

## Introduction

Pro-directed phosphorylation is an important signaling mechanism, which regulates various cellular processes, such as cell proliferation, cell cycle progression, transcriptional regulation, RNA processing and cell differentiation [1], [2]. Peptidyl-prolyl cis/trans somerase NIMA-interacting 1, PIN1, is a key regulator in the postphosphorylation regulatory mechanism, which controls the conformation of pro-directed phosphorylation sites [3], [4]. Consistent with its regulatory function, PIN1 is involved in the process of carcinogenesis. It has been reported that PIN1 is aberrantly over-expressed in some common cancers, such as lung, breast, colon and prostate cancers [5]–[8].

Single nucleotide polymorphisms (SNPs) of PIN1 and cancer risk have been investigated by several studies [9]–[16]. To date, a number of 3 common SNPs of PIN1 have been widely investigated, namely two variants in the PIN1 promoter region: rs2233678 (G>C at nucleotide −842) and rs2233679 (T>C at nucleotide −667) and one SNP in the coding region (rs2233682, G>A; Gln33Gln). Evidence suggested that the rs2233682 polymorphism, the synonymous change of PIN1, did not alter cancer risk [10], [11]. However, the correlation between rs2233678 (−842 G>C) polymorphism and susceptibility to cancer was still inconclusive. Han and colleagues [10] found that the C allele of −842 G>C polymorphism was associated with reduced risk of breast cancer, while Segat and Naidu showed the −842 G>C polymorphism did not affect susceptibility to hepatocellular carcinoma [15] or breast cancer [14]. Thus, it is necessary to ascertain whether the rs2233678 (−842 G>C) polymorphism is associated with altered cancer risk or not. To answer this question, we performed this meta-analysis to provide a more precise estimation of the association and better understand of the relationship between rs2233678 (−842 G>C) polymorphism and cancer risk.

## Results

**Figure 1:**
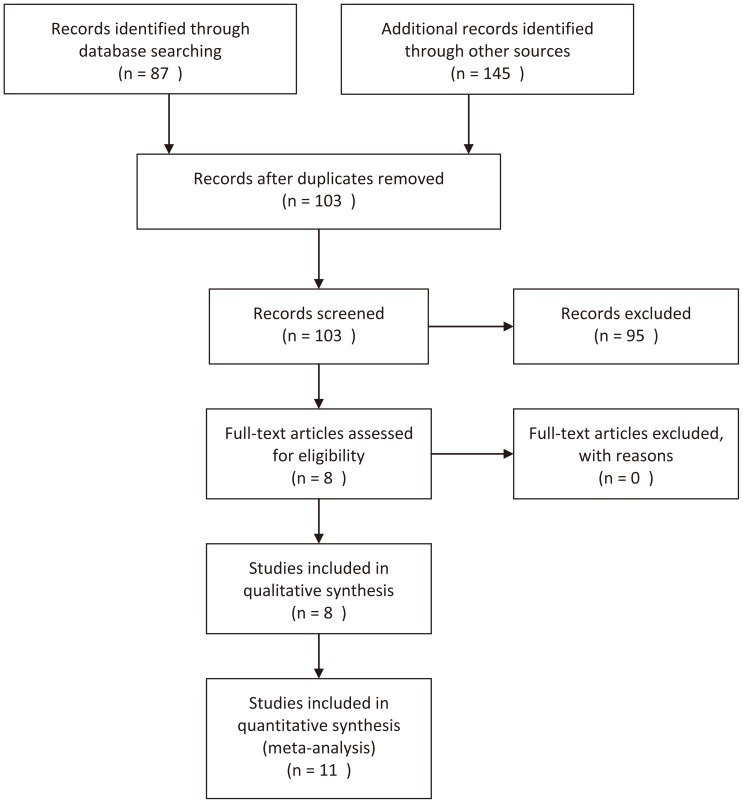
Flow chart of study identification. In the articles by Naidu and Lu, they reported 3 studies and 2 studies separately, respectively, and each of them was treated as an independent study. Thus, a total of 11 studies were included in quantitative synthesis.

There were 87 articles relevant to searching strategy (PubMed: 12; EMBASE: 31; CNKI: 44). The flow chart shown in Figure 1 summarizes the study selection process. In the study by Naidu and colleagues [14], the genotype data were presented separately according to different population (Malays, Chinese and Indians); in the study by Lu et al [12], genotype data were also presented separately according to different study set (test set and validation set). Therefore, we treated them as separate studies. Thus, a total of 11 independent studies [9]–[16] including 4619 cases and 4661 controls were used in this meta-analysis. PIN1 polymorphisms and cancer risk was investigated in 7 kinds of cancer (esophageal carcinoma, laryngeal squamous cell carcinoma, squamous cell carcinoma of the head and neck, hepatocellular carcinoma, breast cancer, lung cancer and nasopharyngeal carcinoma). The eligible studies indentified and main characteristics are listed in Table 1, as well as data of genotype distribution. There were 8 studies of Asian descent and 3 studies of Caucasian descent. Test for Hardy-Weinberg equilibrium (HWE) in the control population was performed for each study, and the genotypes distribution was not in agreement with HWE in one study [15].

**Table 1:** Characteristics of included studies.

Author	Country	Ethnicity	Cancer	ControlSource	Adjusted Factors	Case			Control		
						GG	GC	CC	GG	GC	CC
You Y(2013)	China	Asian	EC	PB	age, sex, BMI, family history of cancer, smoking, drinking status	621	75	3	607	114	8
Lu Y(2012)	China	Asian	NC	HB	age, sex	135	22	21	110	38	8
Cao WP(2012)	China	Asian	LSCC	HB	NA	87	8	0	74	23	3
Lu J(2011,test set)	China	Asian	LC	HB	age, sex, smoking status, alcohol use, family history of cancer	948	103	5	895	154	7
Lu J(2011,validation set)	China	Asian	LC	HB	age, sex, smoking status, alcohol use, family history of cancer	432	67	4	501	117	5
Naidu R(2011,Malay)	Malaysia	Asian	BC	PB	Age	78	28	1	53	24	3
Naidu R(2011,Chinese)	Malaysia	Asian	BC	PB	Age	163	54	2	72	35	4
Naidu R(2011,Indian)	Malaysia	Asian	BC	PB	Age	45	15	1	48	11	2
Han CH(2010)	USA	Caucasian	BC	HB	age, smoking status, alcohol use	358	101	8	336	143	9
Lu J(2009)	USA	Caucasian	SCCHN	HB	age, sex, smoking status alcohol use	838	159	9	794	202	11
Segat L(2007)	Italy	Caucasian	HCC	HB	NA	167	59	2	203	40	7

HB: hospital-based; PB: population-based; EC: esophageal carcinoma; NC: nasopharyngeal carcinoma; LSCC: laryngeal squamous cell carcinoma; LC: lung cancer; BC: breast cancer; SCCHN: squamous cell carcinoma of the head and neck; HCC: hepatocellular carcinoma; NA: not available.

### Main Results

#### −842 G>C polymorphism and cancer risk estimated by genotype distribution data

**Figure 2:**
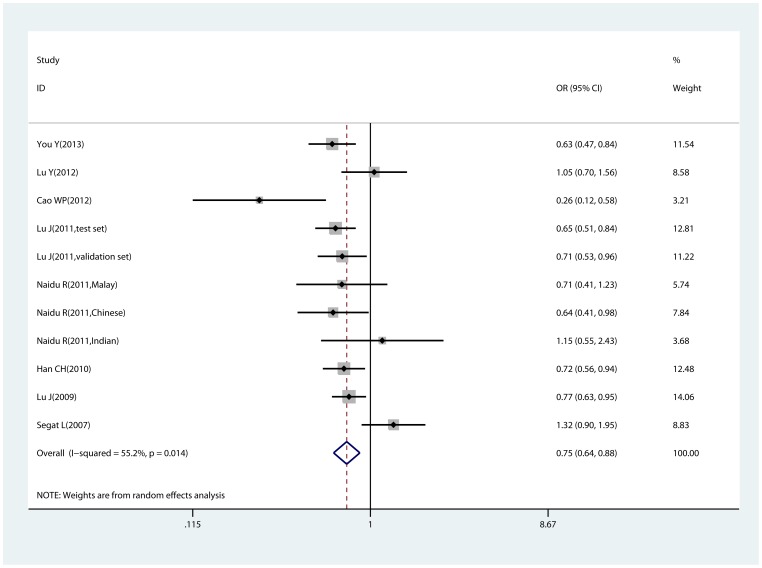
Forest plot of allele comparison (C vs. G estimated by genotype distribution data. Allele comparison calculated with random-effects model. Odds ratio = 0.750, 95% confidence intervals: 0.639–0.880.

Table 2 shows detailed comparison results and heterogeneity among studies. By directly pooling genotype distribution data, in overall comparison, we found that the −842 G>C polymorphism was associated with decreased cancer risk, namely the PIN1 −842 C allele significantly reduced cancer risk compared with the −842 G allele (C vs. G, OR = 0.750, 95% CI:0.639–0.880, P_heterogeneity_ = 0.014, Figure 2). Significant association was also observed in the comparisons of GC vs. GG and CC+GC vs. GG. Subgroup analyses were performed according to ethnicities, sources of control and sample size. No significant association of the −842 G>C polymorphism with cancer risk was observed among Caucasian, while carriers of the C allele showed a lower risk in Asian. The sources of control did not affect pooled results in that both results from population-based or hospital-based studies were roughly consistent. By stratifying studies by sample size (studies of 500 or more participants were classified as large, otherwise were classified as small), we found that large studies provided significant association, while small studies did not found any remarkable differences.

**Table 2:** Meta-analysis results estimated by genotype distribution data.

	C vs. G		CC vs. GG		GC vs. GG		CC vs. GC+GG		CC+GC vs. GG	
	OR(95% CI)	P_het_	OR(95% CI)	P_het_	OR(95% CI)	P_het_	OR(95% CI)	P_het_	OR(95% CI)	P_het_
Overall	0.750(0.639–0.880)*	0.014	0.740(0.515–1.063)	0.252	0.721(0.591–0.880)*	0.003	0.800(0.559–1.146)	0.177	0.725(0.607–0.865)*	0.012
Asian	0.694(0.574–0.839)*	0.087	0.768(0.486–1.212)	0.117	0.641(0.543–0.757)*	0.33	0.849(0.540–1.335)	0.09	0.654(0.559–0.764)*	0.35
Caucasian	0.870(0.645–1.173)	0.029	0.695(0.384–1.261)	0.627	0.926(0.572–1.499)	0.001	0.725(0.401–1.310)	0.483	0.892(0.589–1.353)	0.004
HB	0.770(0.622–0.953)*	0.004	0.900(0.601–1.348)	0.28	0.701(0.535–0.919)*	0.001	0.978(0.656–1.460)	0.19	0.728(0.574–0.924)*	0.003
PB	0.673(0.545–0.831)*	0.505	0.315(0.129–0.769)*	0.925	0.714(0.559–0.910)*	0.378	0.332(0.136–0.808)*	0.952	0.677(0.538–0.853)*	0.425
Large studies	0.704(0.629–0.789)*	0.786	0.706(0.436–1.142)	0.868	0.676(0.597–0.765)*	0.897	0.757(0.468–1.223)	0.855	0.677(0.599–0.765)*	0.868
Small studies	0.802(0.543–1.183)	0.004	0.787(0.454–1.365)	0.048	0.779(0.455–1.333)	<0.001	0.860(0.500–1.479)	0.029	0.789(0.501–1.243)	0.003

OR: odds ratio; P: p value of heterogeneity; HB: hospital-based; PB: population-based;

* significant association.

#### −842 G>C polymorphism and cancer risk estimated by adjusted ORs

**Figure 3:**
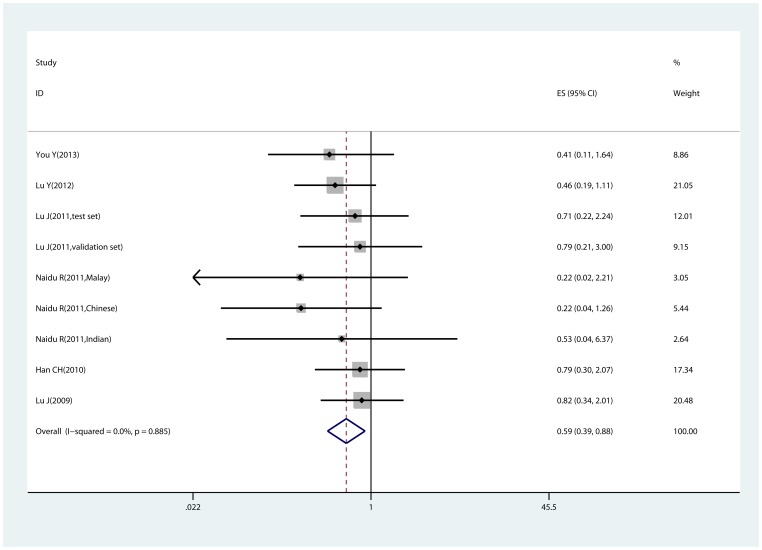
Forest plot of homozygote comparison (CC vs. GG) estimated by adjusted odds ratios. Homozygote comparison calculated with fixed-effects model. Odds ratio = 0.589, 95% confidence intervals: 0.394–0.880.

Table 3 shows the meta-analysis results calculated by adjusted ORs. Consistent with results from genotype data, the −842 C allele of PIN1 was associated with reduced susceptibility to cancer in all three comparisons (homozygote comparison, heterozygote comparison and dominant model), especially in homozygote comparison (CC vs. GG, OR = 0.589, 95% CI:0.394–0.880, P_heterogeneity_ = 0.885; Figure 3), in which no significant association was observed when estimated by genotype distribution data. Additionally, reduced cancer risk were observed in every subgroup, including Caucasian population.

**Table 3:** Meta-analysis results estimated by adjusted odds ratios.

	CC vs. GG		GC vs. GG		CC+GC vs. GG	
	OR(95% CI)	P_het_	OR(95% CI)	P_het_	OR(95% CI)	P_het_
Overall	0.589(0.394–0.880)*	0.885	0.664(0.590–0.747)*	0.527	0.668(0.594–0.751)*	0.638
Asian	0.486(0.292–0.810)*	0.896	0.632(0.542–0.738)*	0.442	0.636(0.545–0.742)*	0.547
Caucasian	0.806(0.419–1.550)	0.956	0.710(0.592–0.850)*	0.55	0.713(0.596–0.853)*	0.6
HB	0.682(0.435–1.069)	0.901	0.665(0.582–0.761)*	0.681	0.678(0.593–0.776)*	0.812
PB	0.327(0.133–0.804)*	0.905	0.660(0.516–0.842)*	0.188	0.636(0.502–0.806)*	0.26
Large studies	0.720(0.442–1.173)	0.938	0.656(0.578–0.745)*	0.682	0.656(0.580–0.743)*	0.663
Small studies	0.386(0.190–0.784)*	0.837	0.715(0.521–0.982)*	0.208	0.768(0.537–1.098)	0.346

OR: odds ratio; P: p value of heterogeneity; HB: hospital-based; PB: population-based;

* significant association.

### Evaluation of Publication Bias, Heterogeneity and Sensitivity

**Figure 4:**
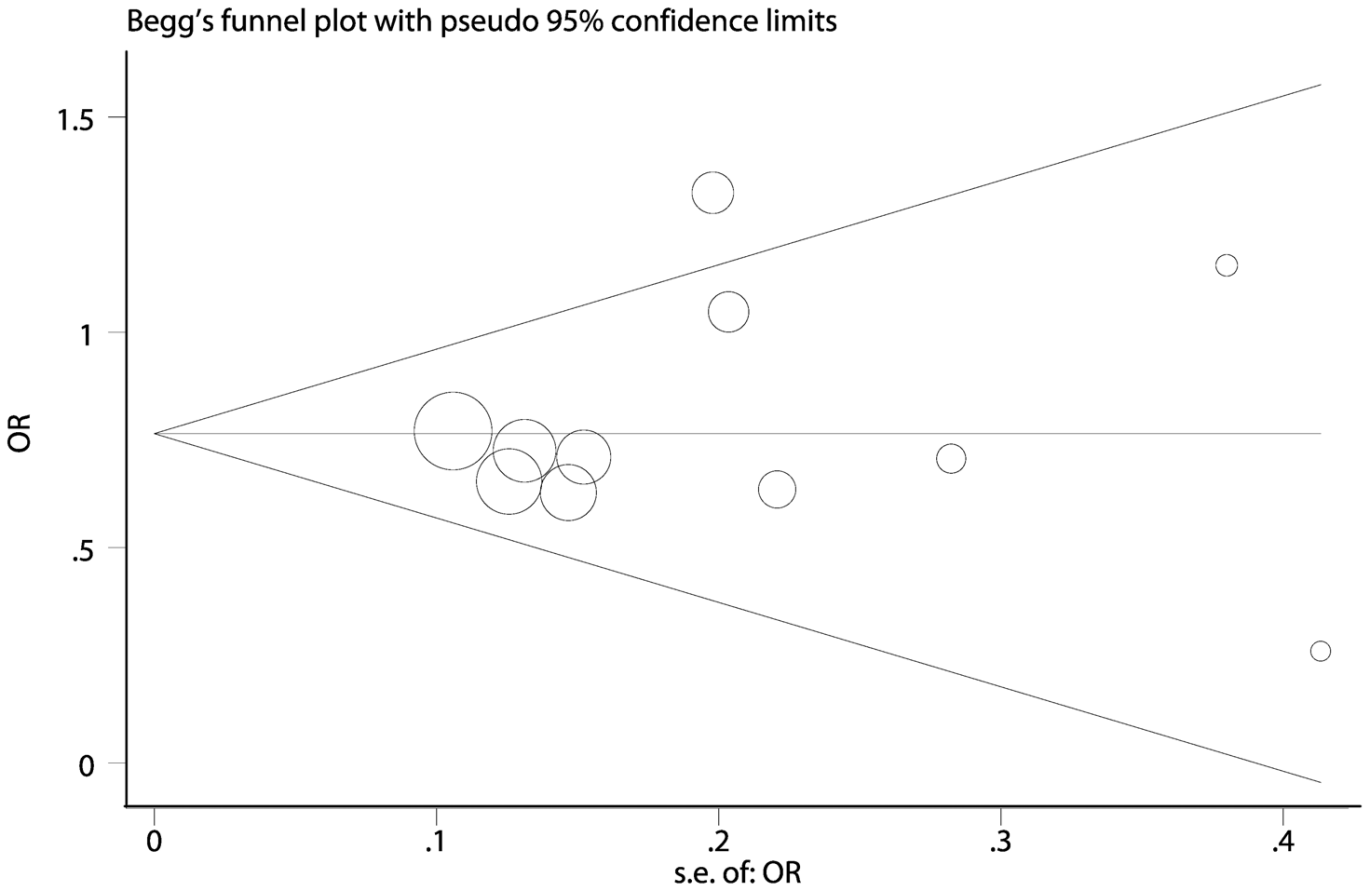
Funnel plot of allele comparison (C vs. G) estimated by genotype distribution data. The circles represent the weight of individual study. Egger’s test, p = 0.604; Begg’s test, p = 1.000.

**Figure 5:**
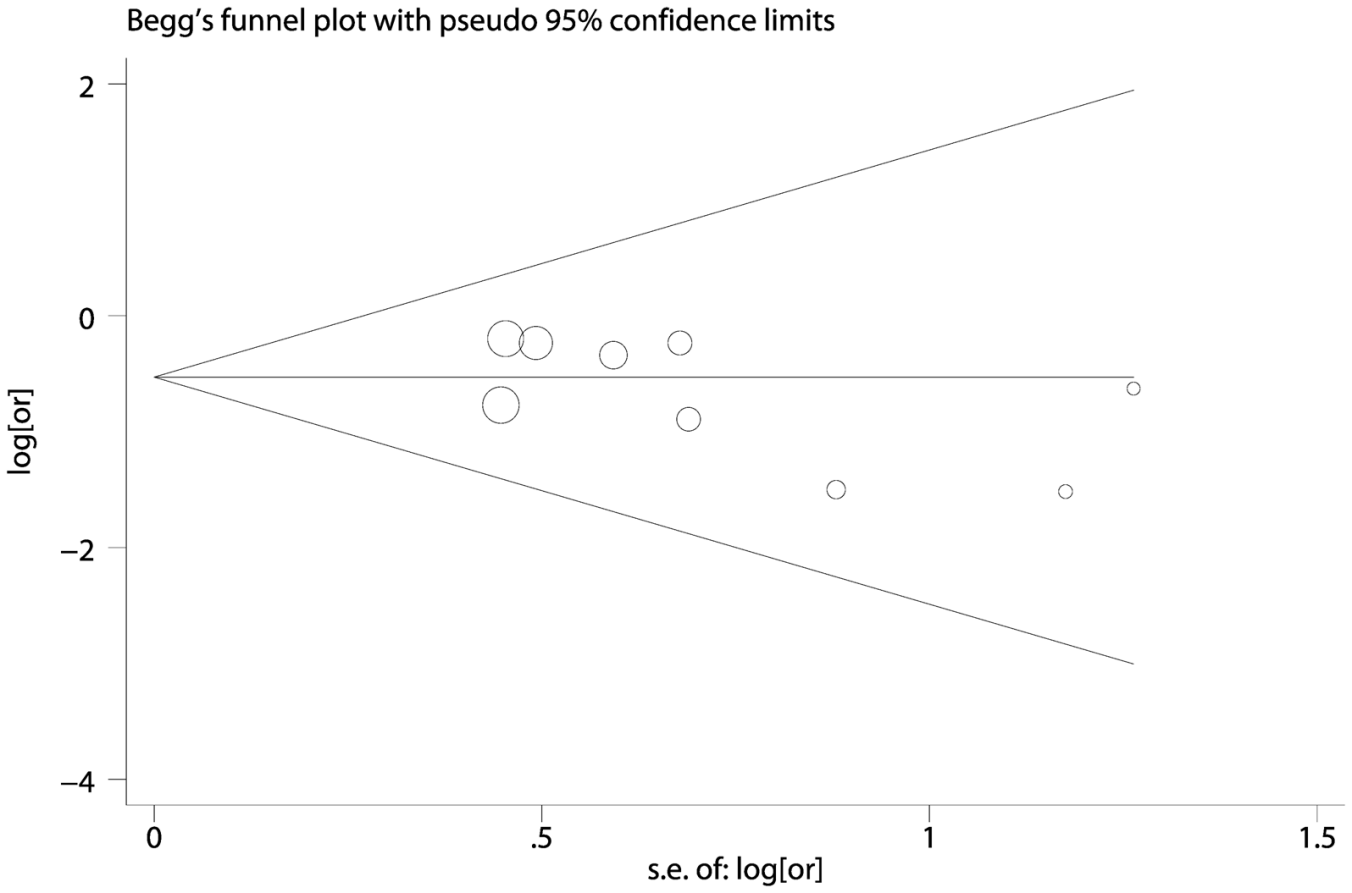
Funnel plot of homozygote comparison (CC vs. GG) estimated by adjusted odds ratios. The circles represent the weight of individual study. Egger’s test p = 0.124; Begg’s test, p = 0.175.

Egger’s test and Begg’s test were performed to assess the publication bias of eligible studies. These tests revealed no evidence of publication bias (C vs. G estimated by genotype distribution data, P_Begg_ = 1.000, P_Egger_ = 0.604, Figure 4; CC vs. GG estimated by adjusted ORs, P_Begg_ = 0.175, P_Egger_ = 0.234, Figure 5). As shown in Table 2, heterogeneity was significant in allele and heterozygote comparison, thus meta-regression was conducted to detect the source of heterogeneity. We found that ethnicities (p = 0.021) and sample size (p = 0.02) but not sources of control (p = 0.069) contributed to heterogeneity. Sensitivity analysis was also performed by omitting one study each time to assess the effect of individual study. No individual study affected pooled results significantly (data not shown).

## Discussion

In this meta-analysis, 11 studies [9]–[16], including 9280 participants, were identified and analyzed. We demonstrated that the rs2233678 (−842 G>C) polymorphism in the PIN1 promoter region was associated with a significantly decreased susceptibility to cancer. This association was observed in both Asian and Caucasian population.

The human PIN1 gene is located on chromosome 19p13, with a promoter region about 1.5 kb. PIN1 belongs to the evolutionarily conserved peptidyl-prolyl isomerase (PPIase) family of proteins [17] that modulates the isomerization of proline amide bonds between the *cis* and *trans* configuration, thereby changing the confirmation of its substrate [2], [18]. PIN1 contains a carboxy-terminal catalytic domain and a conserved WW (Trp-Trp) domain which can change conformation of phosphoproteins by recognizing and binding to specific phosphor-Ser/Thr-Pro motifs [19]. Previous studies have demonstrated that PIN1 regulates numerous oncogenic and tumor suppressor proteins, such as cyclin D1 [20], Cdc27 [21], c-Jun [5], β-catenin [8], Bcl-2 [22], Mytl [23], NFAT [24], CK-2 [25], p53 and p73 [26]. All these proteins contain phosphorylated Ser/Thr-pro motifs and are key regulators of cell cycle or oncogenic and tumor suppressor proteins. Additionally, aberrant expression of PIN1 has been reported in various cancers [5]–[8]. Thus, e evidence suggests that PIN1 plays an important role in the process of carcinogenesis.

The two SNPs (rs2233678, −842 G>C; rs2233679, −667 T>C) occurring in the PIN1 promoter region have been shown to affect the expression level of PIN1. Segat and colleagues found that the −842 CC genotype was significantly associated with lower levels of PIN1 protein compared with the −667 CC genotype in peripheral mononuclear cells of healthy participants [27]. Lu and coauthors also showed that the −842 G allele increased PIN1 expression compared to the −842 C allele in head and neck cancer cell lines [11], indicating that the variant −842 C allele reduced the promoter activity. Considering the oncogenetic role of PIN1 and the altered promoter activity caused by −842 G>C variation, it is reasonable to conclude that the −842 G>C polymorphism in the PIN1 promoter region may alter cancer risk.

In the present meta-analysis, we found that significant heterogeneity was present in heterozygote and allele comparison. By performing subgroup analysis and meta-regression, we found that ethnicities and sample size were responsible for the heterogeneity. This could be explained by that the genetic background, risk factors in life styles, and the environmental factors exposed are different between Asian and Caucasian population. In addition, sensitivity analysis was performed to assess the effect of each individual study, and the results suggested that our meta-analysis was not affected by individual study. Furthermore, no evidence of publication bias was detected, which showed that our results were reliable.

However, our results should be interpreted with caution, since this meta-analysis had some limitations. Firstly, limited by the number of genetic association studies, we did not assess the −842 G>C polymorphism and risk of a certain type of cancer. Since the risk factors of one cancer differ from others, our results could not simply applied to all kinds of cancer. Secondly, sample size of each included studies were relatively small, which may possibly lead to bias though sensitivity analysis, Begger’s test and Egger’s test revealed no significant findings. Thirdly, the genotype distribution in controls did not agree with Hardy-Weinberg equilibrium in one study, which may disturb pooled results. However, when this study was excluded, we still observed a significant association.

To summary, our meta-analysis suggests that the −842 G>C polymorphism is associated with decreased cancer risk. To conform this association, large sample-sized and well-designed case-control studies are warranted.

## Materials and Methods

### Identification of Eligible Studies

This study was carried out and reported in agreement with the PRISMA guidelines for systematic reviews and meta-analyses (supplementary information: Table S1. PRISMA checklist). Eligible case-control studies were extracted by searching databases and manual search of references of relative articles and reviews. In order to identify as many relative articles as possible, PubMed, EMBASE, and China National Knowledge Infrastructure (CNKI) were searched using key words “PIN1”, “polymorphism”, and “cancer”. Alternative spellings of these key words were also considered. There was no limitation of research and the last research was performed on May 2013. References of related studies and reviews were manually searched for additional studies.

### Inclusion and Exclusion Criteria

Studies were selected according to the following inclusion criteria: (1) case-control studies; (2) investigating the association between PIN1 −842 G>C polymorphism and cancer risk; (3) with genotype distribution data to calculate combined ORs and 95% CIs or available adjusted ORs and 95% CIs. Studies without detail genotype distribution data were excluded. Titles and abstracts of searching records were primarily screened and full text papers were further retrieved to confirm eligibility. Two reviewers (Qi Li and Zhao Dong) extracted eligible studies independently according to the inclusion criteria. Disagreement between two reviewers was discussed with another reviewer (Yong Gao) till consensus was achieved.

### Data Extraction

Data of eligible studies was extracted by two reviewers (Qi Li and Zhao Dong) independently with a pre-designed data-collection form. The following data was collected: name of first author, year of publication, country where the study was conducted, ethnicity, cancer types, source of control, Hardy-Winberg equilibrium, number of cases and controls, genotype frequency in cases and controls, adjusted odds ratios (ORs) and 95% confidence intervals (CIs). Different ethnicity descents were categorized as Asian and Caucasian. Eligible studies were defined as hospital-based (HB) and population-based (PB) according to the control source. When Hardy-Winberg equilibrium (HWE) in the controls was tested by chi-square test for goodness of fit. Two reviewers reached consensus on each item.

### Statistical Analysis

The association strength between PIN1 rs3746444 (−842 G>C) polymorphism and cancer risk was measured by OR with 95% CI. The estimates of pooled ORs were achieved by calculating a genotype distribution datagenotype distribution data or adjusted ORs and 95% CIs from each study. A 95% CI was used for statistical significance test and a 95% CI without 1 for OR indicating a significant increased or reduced cancer risk. The pooled ORs were calculated for allele comparison (C versus G), homozygote comparison (GG versus CC), heterozygote comparison (GC versus GG), dominant (CC+GC versus GG) and recessive (CC versus GC+GG) modes, assuming dominant and recessive effects of the variant C allele, respectively. Subgroup analyses were also conducted to explore the effects of confounding factors: ethnicities, sources of control and sample size. Sensitivity analyses were performed to indentify individual study’ effect on pooled results and test the reliability of results.

Chi-square based Q test was used to check the statistical heterogeneity between studies, and the heterogeneity was considered significant when p<0.10 [28]. The fixed-effects model (based on Mantel-Haenszel method) and random-effects model (based on DerSimonian-Laird method) were used to pool the data from different studies. The fixed-effects model was used when there was no significant heterogeneity; otherwise, the random-effects model was applied [29]. Meta-regression was performed to detect the source of heterogeneity and a p<0.05 was considered significant [30].

Publication bias was detected with Begg’s funnel plot and the Egger’ linear regression test, and a p<0.05 was considered significant [31]. All statistical analyses were calculated with STATA software (version 10.0; StataCorp, College Station, Texas USA). And all P values were two-side.

## Supporting Information

Table S1 **PRISMA checklist.**
(DOC)
